# Long-Term Survival of Virulent Tularemia Pathogens outside a Host in Conditions That Mimic Natural Aquatic Environments

**DOI:** 10.1128/AEM.02713-20

**Published:** 2021-02-26

**Authors:** Igor Golovliov, Stina Bäckman, Malin Granberg, Emelie Salomonsson, Eva Lundmark, Jonas Näslund, Joseph D. Busch, Dawn Birdsell, Jason W. Sahl, David M. Wagner, Anders Johansson, Mats Forsman, Johanna Thelaus

**Affiliations:** a Department of Clinical Microbiology, Umeå University, Umeå, Sweden; b Laboratory for Molecular Infection Medicine Sweden, Umeå University, Umeå, Sweden; c Division of CBRN Defence and Security, Swedish Defence Research Agency FOI, Umeå, Sweden; d Pathogen and Microbiome Institute, Northern Arizona University, Flagstaff, Arizona, USA; INRS—Institut Armand-Frappier

**Keywords:** *Francisella tularensis*, tularemia, aquatic environment, biofilms, long-term persistence

## Abstract

Francisella tularensis, the causative agent of the zoonotic disease tularemia, can cause seasonal outbreaks of acute febrile illness in humans with disease peaks in late summer to autumn. Interestingly, its mechanisms for environmental persistence between outbreaks are poorly understood. One hypothesis is that F. tularensis forms biofilms in aquatic environments. We utilized two fully virulent wild-type strains: FSC200 (Francisella tularensis subsp. *holarctica*) and Schu S4 (Francisella tularensis subsp. *tularensis*) and three control strains, the attenuated live vaccine strain (LVS; F. tularensis subsp. *holarctica*), a Schu S4 Δ*wbtI* mutant that is documented to form biofilms, and the low-virulence strain U112 of the closely related species Francisella novicida. Strains were incubated in saline solution (0.9% NaCl) microcosms for 24 weeks at both 4°C and 20°C, whereupon viability and biofilm formation were measured. These temperatures were selected to approximate winter and summer temperatures of fresh water in Scandinavia, respectively. U112 and Schu S4 Δ*wbtI* formed biofilms, but F. tularensis strains FSC200 and Schu S4 and the LVS did not. All strains exhibited prolonged viability at 4°C compared to 20°C. U112 and FSC200 displayed remarkable long-term persistence at 4°C, with only 1- and 2-fold log reductions, respectively, of viable cells after 24 weeks. Schu S4 exhibited lower survival, yielding no viable cells by week 20. At 24 weeks, cells from FSC200, but not from Schu S4, were still fully virulent in mice. Taken together, these results demonstrate biofilm-independent, long-term survival of pathogenic F. tularensis subsp. *holarctica* in conditions that mimic overwinter survival in aquatic environments.

**IMPORTANCE** Tularemia, a disease caused by the environmental bacterium Francisella tularensis, is characterized by acute febrile illness. F. tularensis is highly infectious: as few as 10 organisms can cause human disease. Tularemia is not known to be spread from person to person. Rather, all human infections are independently acquired from the environment via the bite of blood-feeding arthropods, ingestion of infected food or water, or inhalation of aerosolized bacteria. Despite the environmental origins of human disease events, the ecological factors governing the long-term persistence of F. tularensis in nature between seasonal human outbreaks are poorly understood. The significance of our research is in identifying conditions that promote long-term survival of fully virulent F. tularensis outside a mammalian host or insect vector. These conditions are similar to those found in natural aquatic environments in winter and provide important new insights on how F. tularensis may persist long-term in the environment.

## Abstract (precis)

Tularemia, a disease caused by the environmental bacterium Francisella tularensis, is characterized by acute febrile illness. F. tularensis is highly infectious: as few as 10 organisms can cause human disease. Tularemia is not known to be spread from person to person.

## INTRODUCTION

Tularemia is a widely distributed zoonotic disease in the Northern Hemisphere caused by the bacterium Francisella tularensis. Human disease is mainly associated with strains from two subspecies, which have different geographical distributions. Francisella tularensis subsp. *holarctica* is found throughout the Northern Hemisphere, whereas Francisella tularensis subsp. *tularensis* is found only in North America. In humans, infections with F. tularensis are acquired from the environment via the bite of blood-feeding arthropods, ingestion of infected food or water, direct contact with infected animals, or inhalation of aerosolized bacteria. Infection can lead to acute febrile illness, and arthropod-related transmission peaks in the summer and autumn of outbreak years. *Francisella* is a large and diverse genus that includes environmental species of minor clinical relevance, including opportunistic pathogens such as Francisella novicida, F. hispaniensis, and F. philomiragia, which cause disease only in immunocompromised humans (1–6). F. tularensis is one of the most infectious pathogenic bacteria known; as few as 10 organisms can cause human infection by inoculation of the skin or inhalation of aerosolized bacteria (7), and it is regarded as a potential biological weapon and bioterrorism agent (8, 9). Naturally occurring F. tularensis causes tularemia in mammals, and more than 250 species have been reported to be naturally infected by F. tularensis (10). All human cases are acquired directly from the environment, and disease events in humans are often associated with zoonotic events in the environment. Predicting outbreaks of tularemia is extremely complex because of the numerous species described as susceptible to this disease, lack of knowledge of the complete life cycle of the bacterium, and the unknown natural reservoir of the bacterium. However, the capacity of F. tularensis to remain highly pathogenic in mammals is likely important to its natural life cycle.

F. tularensis appears to persist in the environment outside infected mammals and arthropods but, despite more than 100 years of research, little is known about the specific mechanisms of this environmental persistence (11). Human tularemia is typically characterized by irregular outbreaks that can be separated by several years within a small geographical region (disease focus). Previous studies have revealed persistence of the bacterium outside mammalian hosts or arthropod vectors in water and sediment for at least 16 months (12). In an area of Sweden where tularemia is endemic, F. tularensis subsp. *holarctica* DNA was detected by PCR in sediment and water samples collected during both outbreak and nonoutbreak years (13). In addition, genomic studies have confirmed the early findings of environmental persistence in disease foci from the early and mid-1900s (14, 15) and also confirmed that these foci have persisted to the present in both Western Europe (16) and Ukraine (17).

Persistence of F. tularensis in nature outside a host would require the ability to survive periods of severe nutrient limitation and variable temperatures. The ability to survive extended periods of starvation or other forms of environmental stress may be potentially conferred by symbiotic or parasitic interactions with protozoa (18–22) and/or associations with arthropod species (23, 24). It has also been hypothesized that F. tularensis forms biofilms, which could enhance persistence in the aquatic environment (25), consistent with the environmental lifestyle of many other bacteria. In laboratory studies performed in rich nutrient medium and at close to body temperature, isolates of F. novicida, *F. philomiragia*, F. tularensis subsp. *holarctica*, and F. tularensis subsp. *tularensis* have all been induced to form biofilms on the wells of plastic culturing plates (26–32).

Lower temperatures and other environmental parameters may be favorable for increased survival in F. tularensis. Cold water contaminated by carcasses or excreta of infected animals can remain infectious for as long as 10 weeks (12, 33). It also has been suggested that oceanic salt sprays may promote viability of F. tularensis in water (34). Berrada and Telford (34) utilized sterilized fresh and brackish water samples from Martha’s Vineyard (Massachusetts, USA) that were incubated at 20°C and found that the longest survival times of both F. tularensis subsp. *tularensis* (32 days) and F. tularensis subsp. *holarctica* (live vaccine strain [LVS], 42 days) occurred in brackish water (3.6% NaCl), with an intermediate survival time in physiological saline solution (0.85% NaCl) and the lowest survival time in freshwater. Additionally, early studies on factors that enhance F. tularensis spp. growth in culture noted that optimal growth could be obtained at 1 to 2% NaCl in culture medium (35). Also, the pH for optimal growth of F. tularensis subsp. *holarctica* is in the range of 5.8 to 6.3 (36), but both F. tularensis subsp. *tularensis* and F. tularensis subsp. *holarctica* show relatively high survival following temporal acid treatment (37, 38), and low-nutrient preadaptation in natural water resulted in even greater acid resistance (38).

In this study, we investigated the survival of F. tularensis at low temperatures in low-nutrient water, and our findings lead us to challenge the hypothesis that fully virulent F. tularensis strains form biofilms under conditions that resemble environments where the bacteria may persist in nature. We documented that the combination of low temperature and physiological saline (0.9% NaCl) increased F. tularensis survival times substantially compared with earlier findings that examined only the effects of saline at higher temperatures.

## RESULTS

### F. tularensis strains do not form biofilms in low-nutrient water at either 20°C or 4°C.

**FIG 1:**
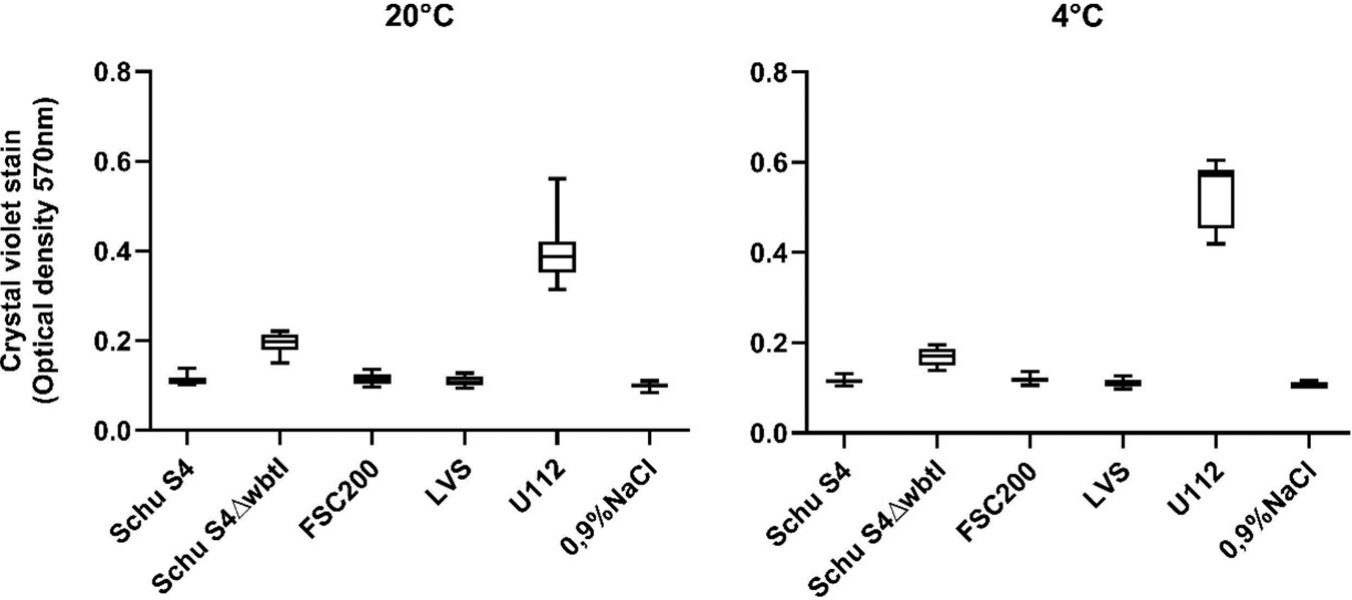
Biofilm formation of *Francisella* strains at 20°C (left) and 4°C (right) measured over time. Bacterial strains were incubated in a saline solution (0.9% NaCl), and biofilm formation was quantified using crystal violet stain and optical density measurement performed at 2, 3, 4, 6, 8, 10, 12, and 24 weeks incubation. Box plots present the median, quartile, minimum, and maximum of measurements. All data from the 2-, 3-, 4-, 6-, 8-, 10-, 12-, and 24-week measurements are presented in one whisker box plot per strain.

The only strains that developed biofilm during incubation in the saline solution (0.9% NaCl), as demonstrated by crystal violet staining, were F. novicida U112 and the Schu S4 Δ*wbtI* mutant (Fig. 1). These strains developed biofilms at the first sampling, performed at 2 weeks, which were quantified by measurement of optical density at 570 nm (OD_570_) as 0.57 ± 0.04 for U112 and 0.17 ± 0.02 for the Schu S4 Δ*wbtI* mutant in the 4°C incubations (Fig. 1). In a hierarchical linear mixed model (LMM), biofilm formation by U112 obtained a corresponding coefficient estimate of 0.43 (95% confidence interval [CI], 0.377 to 0.486; *P* < 1.0E−16) (see Fig. S1 in the supplemental material), whereas Schu S4 Δ*wbtI* resulted in an estimate of 0.059 (95% CI, 0.008 to 0.110; *P* = 0.038). Both comparisons were made relative to the negative control with saline solution (0.9% NaCl), suggesting that biofilm formation by the U112 strain is predicted to be 0.43 crystal violet stain assay unit above the negative control, on average.

The amount of biofilm formed by the Schu S4 Δ*wbtI* strain was constant throughout the experiment, with no significant increase in crystal violet staining from sampling week 2 to week 24 (model estimate, 0.003; standard deviation, 0.006). For strain U112, the amount of biofilm decreased throughout the sampling period (estimate, −0.016; standard deviation, 0.006), suggesting that the biofilm would be reduced on average by 4% each week (if all other conditions were unchanged). At the 24-week sampling, the U112 biofilm in the 4°C incubation was at an optical density of 0.43 ± 0.09 in the crystal violet stain. There was no significant difference in the amount of biofilm formed by U112 and Schu S4 Δ*wbtI* at 4°C compared to the 20°C incubation (estimate, 0.0; 95% CI, −0.013 to 0.013; *P* = 1.0).

F. tularensis strains Schu S4 and FSC200 and the LVS displayed no biofilm formation at either 20°C or 4°C. The crystal violet stain assay results for these strains were not significantly different from that of the negative control with saline solution (0.9% NaCl) and no bacteria (estimate, 0; *P* = 1.0) (see Fig. S1).

**FIG 2:**
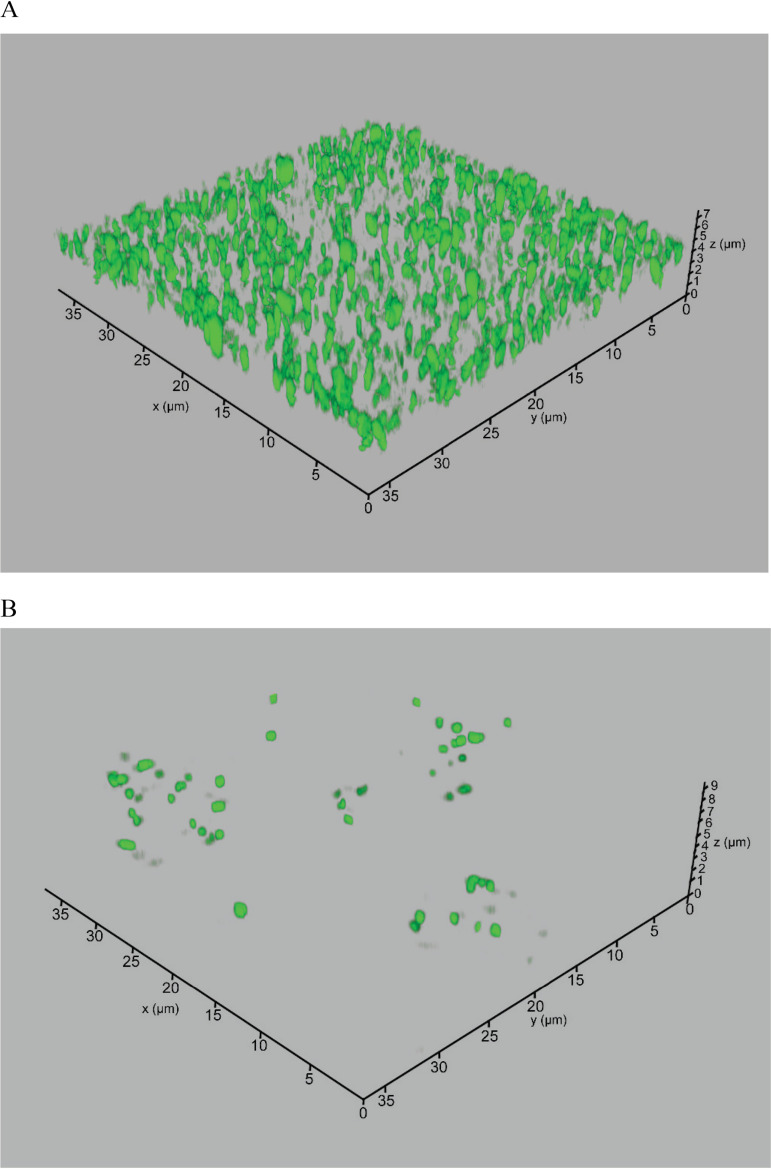
Confocal laser scanning microscopy of U112pKK289Kan-*gfp* (A) and LVSpKK289Kan-*gfp* (B), expressing GFP, after 1 week in 0.9% NaCl in 20°C.

Furthermore, biofilm formation by green fluorescent protein (GFP)-labeled U112 was visualized with confocal microscopy (Fig. 2). Three-dimensional images of 1-week incubations detected the formation of biofilm in U112, with the thickness of attached cells ranging from 0.5 to 4 μm. However, the LVS did not form biofilm, as demonstrated by the crystal violet stain (Fig. 1), and confocal microscopy of a GFP-labeled LVS showed that very few cells were attached to the surface (Fig. 2).

### All strains exhibited increased survival at 4°C compared to 20°C.

**FIG 3:**
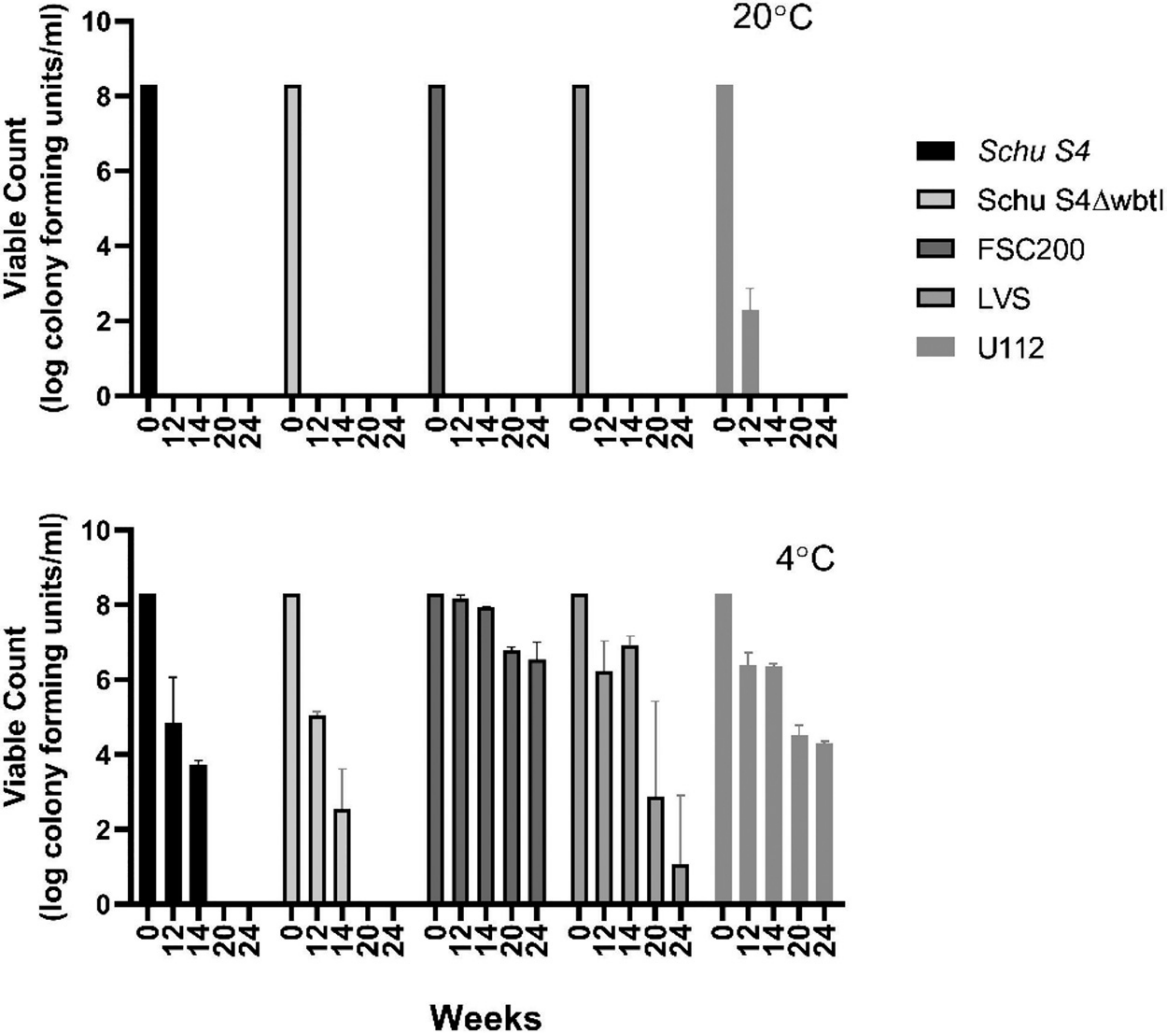
Viable counts of planktonic bacteria in the biofilm assay over time (0, 12, 14, 20, and 24 weeks). *Francisella* strains were incubated in 0.9% NaCl at 20°C and 4°C. Columns show means for six replicates (except those for FSC200 and the LVS at 24 weeks, which show means for three replicates), and error bars represent standard deviations. Absence of bars indicates no CFU.

Viable-count analysis of planktonic cells in the biofilm incubation at 20°C showed a loss of viability by 12 weeks for all strains studied (Fig. 3), except for strain U112, which retained a population of viable cells at 2 × 10^2^ CFU/ml after 12 weeks. After 14 weeks in 20°C, none of the bacterial strains were culturable.

In contrast, all strains retained viability for 14 weeks at 4°C (Fig. 3), irrespective of whether they formed biofilms. Interestingly, Schu S4 lost viability between 14 and 20 weeks, whereas FSC200, the LVS, and U112 were still viable after 24 weeks in the saline solution at 4°C. During the 6-month incubation period, FSC200 showed only a 2-fold log reduction in viable counts, from 1 × 10^8^ CFU/ml to 1 × 10^6^ CFU/ml (Fig. 3).

### F. tularensis subsp. *holarctica* but not F. tularensis subsp. *tularensis* strains maintain virulence after 24 weeks in 4°C low-nutrient water.

**FIG 4:**
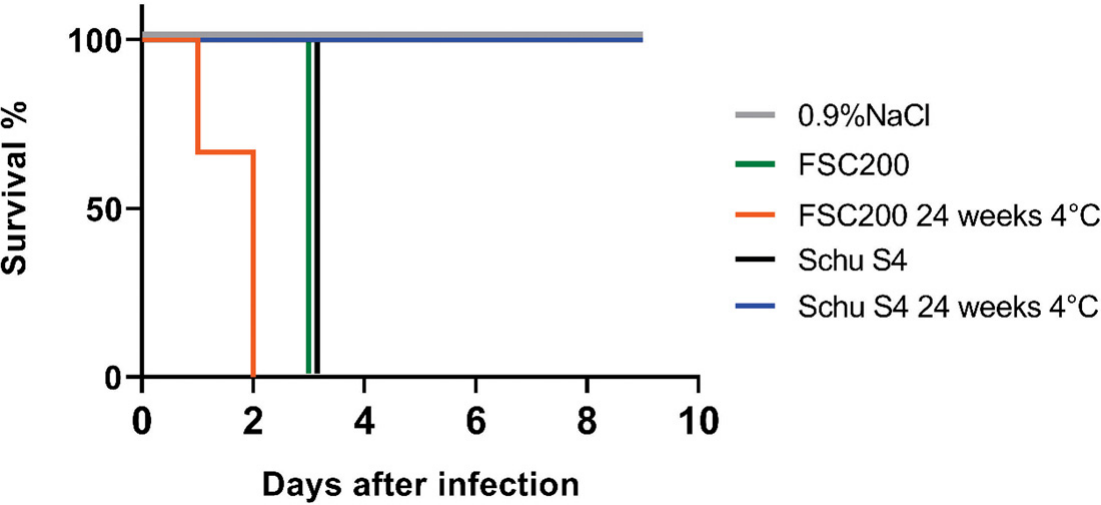
Virulence of Francisella tularensis FSC200 (3 mice) and Schu S4 (3 mice) populations, incubated at 4°C for 24 weeks, in the C57BL/6J mouse model. FSC200 and Schu S4 from overnight cultures and an infectious dose of 10^2^ CFU were used as a control (3 mice for each strain) and 0.9% NaCl was used as a negative control (2 mice).

Mice infected with the FSC200 sample that had been incubated at 4°C for 24 weeks showed symptoms of disease at day 1 or 2 postinfection (Fig. 4). This confirms that the surviving FSC200 population remained highly virulent after incubation at 4°C for 24 weeks. Based on the viable-count analysis of FSC200 incubated at 4°C for 24 weeks (Fig. 3), the mice received an infectious dose of 1 × 10^5^ CFU. In contrast, mice infected with the Schu S4 sample incubated in 4°C for 24 weeks did not show any symptoms of disease during the 10-day study, consistent with a lack of viability (no CFU) observed for these strains past 20 weeks (Fig. 3). Viable-count analysis confirmed the presence of F. tularensis in spleens from mice infected with FSC200 incubated in 4°C for 24 weeks but not in those from mice infected with Schu S4 incubated in 4°C for 24 weeks (data not shown). The control mice that were infected with ∼10^2^ CFU of FSC200 and Schu S4 (grown overnight on supplemented GCII agar) showed symptoms of disease at day 3 postinfection (Fig. 4).

## DISCUSSION

We identified two key insights in the long-term survival of fully virulent strains of F. tularensis outside hosts in the type of nutrient-limited aquatic conditions they would be expected to encounter in nature. First, the long-term survival under these conditions of pathogenic F. tularensis strains known to cause disease in humans does not appear to be dependent on intrinsic biofilm formation. Of course, this does not exclude the possibility that in the environment, F. tularensis survival is potentially enhanced by biofilms produced by other organisms. Second, survival of planktonic *Francisella* cells in nutrient-limited aquatic conditions is greatly extended at lower temperatures. In particular, the planktonic survival of F. tularensis subsp. *tularensis* for over 3 months and F. tularensis subsp. *holarctica* for over 6 months at lower temperatures suggests a critical factor which could allow viable F. tularensis, in general, to overwinter in nutrient-limited aquatic environments. The differences observed between the two subspecies lend support to an idea originally developed by Jellison (39) that F. tularensis subsp. *holarctica* might be more adapted to survival in aquatic environments, such as streams, ponds, lakes, and rivers, whereas F. tularensis subsp. *tularensis* may be less adapted to long-term survival in aquatic conditions.

The only strain that produced significant levels of biofilm under the conditions utilized in this study (i.e., cold and low-nutrient water) was strain U112, which corroborates previous studies that suggest that biofilm formation is an environmental survival mechanism for F. novicida (32). Originally, F. novicida was isolated from a turbid water sample (1), and it has since been detected in brackish and saltwater sources, but no association with arthropod vectors or animals has been demonstrated in nature (1, 23, 40–42). Compared with F. tularensis, the genome of F. novicida is larger and more complex; it shows a higher degree of metabolic competence, higher levels of recombination, and *dN*/*dS* ratios (ratios of nonsynonymous to synonymous substitutions) consistent with a free-living environmental niche (43, 44). Several studies have contributed to the characterization of F. novicida biofilm formation and composition, revealing that the biofilm matrix consist of nucleic acids, proteins, exopolysaccharides (including cellulose), and polymeric β-1,4-linked *N*-acetylglucosamine (26, 27, 29, 31, 32). A gene cluster present in F. novicida but absent in F. tularensis is involved in the synthesis and degradation of the secondary messenger molecule c-di-GMP, which regulates the switch from a free-living planktonic lifestyle to a biofilm-associated lifestyle (45). High c-di-GMP levels program F. novicida to produce biofilm and inhibit virulence (45), thus promoting survival outside the host. This c-di-GMP signaling cascade is missing in F. tularensis, which is consistent with the concept that F. tularensis is a highly niche-restricted pathogen (43). In F. tularensis, loss of the ability to produce a functional lipopolysaccharide (LPS) O-antigen due to a mutation in the *wbt* locus results in enhanced biofilm formation in culture medium at 37°C (31). Accordingly, the only F. tularensis strain where biofilm formation could be detected under the conditions used in this experiment was the Schu S4 Δ*wbtI* mutant, which has a dysfunctional LPS O-antigen; however, this mutant is completely avirulent (46, 47).

F. tularensis is well known for its persistence in water. Parker et al. (12) showed persistence in water and sediment for at least 16 months in the Gird Creek area (Montana, USA). However, a high level of variation exists in the length of time that F. tularensis persists under laboratory conditions. In a sample of naturally contaminated water from Gird Creek stored at 7°C, the pathogen survived for a shorter time, at least 23 days but not more than 35 days (12). Naturally contaminated mud samples originating from Cattail Creek (Montana, USA) were stored under the same conditions but gave varying results, with persistence lasting throughout a 4- to 10-week period (12). Dearmon et al. (48) investigated survival in F. tularensis liquid cultures after storage at constant temperatures (37, 26, 15, 3, and 0°C) for 1 to 111 days and found that long-term survival through the entire time period occurred only at the lowest temperatures (3°C and 0°C), with the highest proportions of culturable cells at 3°C. However, it should be noted that both studies (12, 48) were performed before the division of F. tularensis into subspecies came into practice, and it is not clear if F. tularensis subsp. *holarctica* and/or F. tularensis subsp. *tularensis* was under investigation in these studies. More recent studies have documented that F. tularensis subsp. *holarctica* remains viable for 21 to 70 days at 8°C in sterile tap water (49, 50) and 28 days in low-nutrient sterilized lake water incubated at 20°C (20). Other physical water parameters, such as salinity and pH, may also influence survival of the bacterium. In our experimental setup, we chose to use intermediate conditions for both salinity and pH (0.9% NaCl and pH 6.0).

Carcasses of wild animals deceased from tularemia and with high bacterial burdens, such as voles and beavers, can contaminate local surface waters (12, 15, 51–54). In line with this, F. tularensis subsp. *holarctica* has recently been shown to be equipped with a mechanosensitive channel that protects it from osmotic variations in its life cycle, such as that encountered when transitioning from a mammalian host to freshwater or drinking water (55). It is not known whether F. tularensis subsp. *tularensis* possesses this ability, but if it does not, this difference may account for the greater association of F. tularensis subsp. *holarctica* strains with water (56). In Turkey, oropharyngeal waterborne tularemia caused by contamination of water by small rodents deceased from infections with F. tularensis subsp. *holarctica* is frequently reported. The greater part of these outbreaks have occurred in late fall, winter, or early spring (57–59), which suggests that survival in lower water temperatures is significant for the epidemiology of F. tularensis subsp. *holarctica*. In Sweden, where humans predominantly develop ulceroglandular tularemia from bites of mosquitoes infected with F. tularensis subsp. *holarctica* (60–62), it is hypothesized that mosquitos may acquire the bacterium from the ephemeral waters that constitute the habitat of mosquito larvae. These temporary water pools occur in early spring during snowmelt and later in the year during periods of heavy rainfall and are prone to alternate between dilution by rain and an increase in salinity due to evaporation. Field-collected mosquito larvae reared in such temporary waters, as well as mosquito larvae experimentally exposed to F. tularensis subsp. *holarctica* during development in a laboratory setting, have been documented to harbor the bacteria as adults (63–65). Thus, F. tularensis (F. tularensis subsp. *holarctica* in particular) seems well adapted to survive the transition from mammalian hosts to cold-water conditions found in the environment that are prone to large variation in osmolarity.

It is important to note that this study considered only axenic conditions for bacterial biofilm formation and survival and that conditions in natural waters with a high background of competing and predatory microorganisms are bound to play an important role in bacterial persistence in water. Interestingly, Buse et al. (66) observed growth of F. tularensis subsp. *tularensis* (strain Schu S4) but not F. tularensis subsp. *holarctica* (the LVS and strain IN99) among a high background of competing microorganisms in well water storage experiments when shifted to a 37°C incubation. This suggest that in these complex environments, F. tularensis subsp. *holarctica* remains nonactive in response to a temperature shift, whereas F. tularensis subsp. *tularensis* is more opportunistic or competitive and prone to induce growth. As shown for several species of aquatic bacteria, there is a competitive advantage in inactivity (or entering a quiescent state) (67, 68). Quiescent bacteria in cold-water conditions have been shown to experience lower biological competition than actively growing cells (69).

Our findings suggest that the planktonic survival of F. tularensis subsp. *holarctica* for over 6 months in cold water may be a critical mechanism by which viable and fully virulent cells overwinter. This suggests the possibility of survival of F. tularensis in the environment between host-associated replication events. The maximum density point of freshwater is at approximately 4°C, and this property of freshwater ensures that the bottom of a body of water will remain at low temperature, yet unfrozen, during the winter period. This type of thermal stratification is usually seasonal in the Northern Hemisphere, where F. tularensis occurs. Mixing of the water layers during spring turnover would then lead to contact of the bacteria with susceptible animals, either directly by intake of water or indirectly through bridging vectors associated with water. These results are compatible with a decay model of survival in the environment in which, after a certain time has passed (time lapse varying with ecosystem and environmental conditions as well as presence of hosts, vectors, and reservoirs), the bacteria will go extinct unless a susceptible host can be infected.

## MATERIALS AND METHODS

### Bacterial strains and growth conditions.

All *Francisella* strains used in this study (Table 1) were cultured on GCII agar (chocolate agar) containing 1% hemoglobin and 1% IsoVitaleX (70). The cultures were incubated at 37°C in 5% CO_2_ if not stated otherwise. Laboratory work involving virulent strains was performed in a biosafety level 3 (BSL3) laboratory.

**TABLE 1:** *Francisella* strains and constructs used in the study

Strain/construct	Subspecies	Description	Source and/or reference
U112	F. novicida	Identified as FSC040 here and in previous publications	Water, 1950, Utah (1)
FSC200	F. tularensis subsp. *holarctica*	Wild-type strain	Human ulcer, 1998, Sweden (76)
LVS	F. tularensis subsp. *holarctica*	Live vaccine strain; identified as FSC155 here and in previous publications	USAMRIID
Schu S4	F. tularensis subsp. *tularensis*	Wild type strain. Identified as FSC237 here and in previous publications	Human ulcer, 1941, Ohio (77)
Schu S4 Δ*wbtI*	F. tularensis subsp. *tularensis*	Mutant of FSC237 characterized by loss of the long-chain O-polysaccharide	47
LVSpKK289Kan-*gfp*	F. tularensis subsp. *holarctica*	FSC155 expressing GFP in *trans*	This study
U112pKK289Kan-*gfp*	F. novicida	FSC040 expressing GFP in *trans*	This study
pKK289Kan-*gfp*		*groELS* promoter; Ft ori; p15A ori; GFP; Kan^r^	71

### Construction of a F. novicida strain expressing GFP.

The pKK289Kan-*gfp* plasmid (71) was introduced into U112 and the LVS by electroporation as previously described (72). The resulting strains, U112pKK289Kan-*gfp* and LVSpKK289Kan-*gfp*, expressing GFP in *trans*, were verified by selection on GCII agar plates supplemented with kanamycin.

### Microtiter plate biofilm quantification assay and viable count.

Bacterial strains were grown overnight as described above. The bacteria were then harvested from the surface of GCII agar plates and suspended in saline solution (0.9% NaCl, pH 6) at a concentration of 10^8^ CFU/ml, as determined by measurement of optical density at 540 nm (OD_540_). Six replicate aliquots of 200 μl from each concentrated inoculum were placed in standard 96-well plates and sealed. One 96-well plate was prepared for each sampling time point and duplicated for the two incubation temperatures, 20°C and 4°C. Planktonic growth at different temperatures was assessed with viable counts at weeks 0, 12, 14, 20, and 24 by plating 100 μl onto supplemented GCII agar plates from 10-fold serial dilutions in 1× phosphate-buffered saline (1× PBS).

To measure biofilm formation, planktonic bacteria (at weeks 2, 3, 4, 6, 8, 10, 12, and 24) were aspirated and wells were washed three times with 1× PBS to remove all remaining nonadherent cells. Plates were incubated for 1 h at 37°C, stained with 200 μl of 0.2% (wt/vol) crystal violet/well for 15 min, and washed 4 or 5 times with PBS. Plates were air dried, after which the dye bound to the adherent cells was resolubilized by the addition of 200 μl of 95% ethanol. The optical density of each well was measured at 570 nm using a microtiter plate reader.

### Confocal microscopy.

Biofilm presence and thickness were evaluated using two GFP-expressing strains (U112pKK289Kan-*gfp* and LVSpKK289Kan-*gfp*) (Table 1). Bacteria were grown on solid agar as described above and suspended in 0.9% NaCl at a concentration of 10^9^ CFU/ml, as determined by optical density (OD_540_). Bacterial suspensions of 150 μl were incubated on 8-well chamber slides (ibidi, Germany). After 1 week of incubation at room temperature, wells were washed three times with 1× PBS. Confocal microscopy was performed on a Leica SP8 inverted confocal system (Leica Microsystems) equipped with a HC PL APO 63×/1.40 numerical aperture oil immersion lens. Scanning was performed in line-by-line sequential mode. Images were captured and processed using LasX (Leica Microsystems) software.

### Virulence in mice.

C57BL/6J female mice, aged 7 to 10 weeks (Scanbur), were used for virulence studies. Mice were housed under conventional conditions, given food and water *ad libitum*, and allowed to acclimatize before infection. The study was approved by the Local Ethical Committee on Laboratory Animals in Umeå, Sweden (A43-2018). Mice were injected intraperitoneally (i.p.) with 100 μl of the FSC200 and Schu S4 cells stored for 24 weeks at 4°C (in replicates of three mice per bacterial strain). As a positive control, mice were injected via the i.p. route with FSC200 (*n* = 2; 600 CFU each) and Schu S4 (*n* = 3; 100 CFU each) cells grown on GCII agar containing 1% hemoglobin and 1% IsoVitaleX, as described above. For negative controls, two mice were injected with 100 μl of 0.9% NaCl. Mice were observed for the weight reduction that precedes the visible symptoms of murine tularemia (i.e., >1.2 g of weight reduction within a single 24-h period). Moribund mice were euthanized immediately, and all remaining mice were euthanized after 10 days. All virulence tests in mice were performed under BSL3 conditions. The presence of *Francisella* in euthanized mice was confirmed by serial dilutions of homogenized spleens plated on agar plates (as described above).

### Statistical analysis.

To assess the differences in biofilm production, a hierarchical linear mixed model (LMM) was fitted to the biofilm measurements, with crystal violet stain measurements as the response variable and temperature and strain as fixed effects. The weekly effect for each strain was included as random slope in the model to account for deviations across the sampling period (i.e., a linear growth or decay in biofilm production of each strain), while the “interaction week × strain” effect was included as a random intercept in the model to account for replicate dependencies. The R package lme4 (73) was used to fit the LMM to the data. Default values of the parameters controlling convergence of the glmer function in the lme4 package were used. *P* values of fixed effect estimates were calculated via a *t* test using Satterthwaite's degrees-of-freedom method implemented in the R package lmerTest (74). Confidence intervals (CI) were calculated using the Wald method. The coefficient estimates plot was created using the R package sjPlot (75).

## Supplementary Material

Supplemental file 1
