# Supplementary material for: Long-Term Survival of Virulent Tularemia Pathogens outside a Host in Conditions That Mimic Natural Aquatic Environments
Source: Appl Environ Microbiol. 2021 Feb 26;87(6):e02713-20. doi: 10.1128/AEM.02713-20 (PMC8104992; doi:10.1128/AEM.02713-20)
Supplement: Supplemental file 1 [file AEM.02713-20-s0001.pdf]

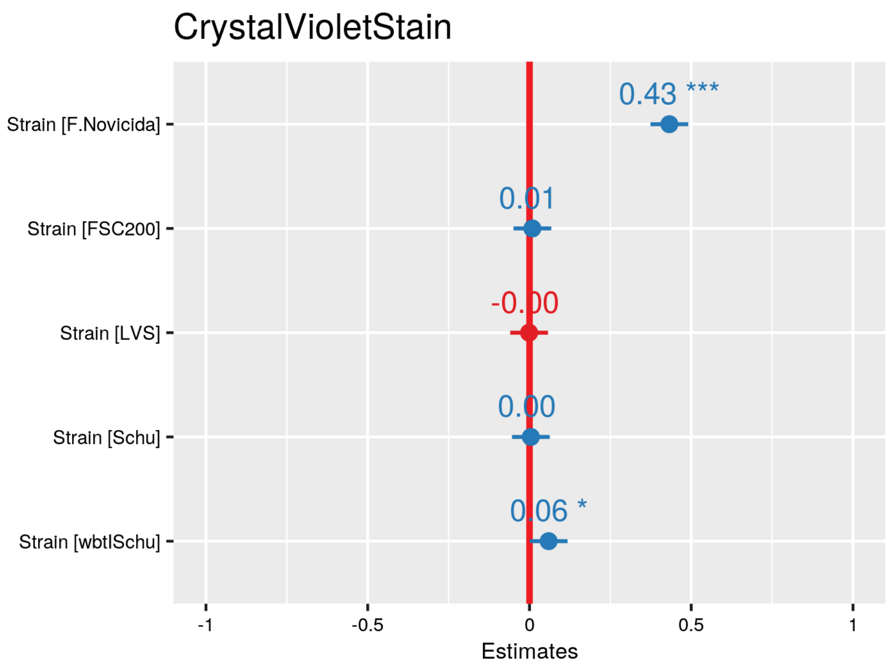

Supplementary Figure 1. Plot of the coefficient estimates of biofilm formation from the Crystal Violet stain assay. The strain effect on the Crystal Violet stain assay by *Francisella* spp. strains (*F. novicida* U112, FSC200, LVS, Schu S4 and Schu S4 Δ*wbtI* mutant) is calculated via comparison to the negative control (saline solution, 0.9% NaCl). Point estimates (dots) for each strain are highlighted in the figure together with 95% confidence interval (lines). Blue correspond to negative point estimates and red corresponds to positive point estimate values. * Significance value p = 0,05. *** Significance value p = 0,001.
